# Effect of Polyethylene Glycol and Activated Carbon Macroparticles on Thermal Conductivity of Paraffin Wax for Thermal Storage Applications

**DOI:** 10.3390/polym14194181

**Published:** 2022-10-05

**Authors:** Lwin Phone Myat, Muhammad Shakeel Ahmad, Indra Neel Pulidindi, Hamed Algarni, Laveet Kumar, Abul Kalam, S. Wageh, Adarsh Kumar Pandey, Altaf Akbar, Jeyraj Selvaraj

**Affiliations:** 1 Higher Institution Centre of Excellence (HICoE), UM Power Energy Dedicated Advanced Centre (UMPEDAC), Level 4, Wisma R&D, University of Malaya, Jalan Pantai Baharu, Kuala Lumpur 59990, Malaysia; 2 Jesus’s Scientific Consultancy of Industrial and Academic Research (JSCIAR), Chennai 600 113, India; 3 Research Center for Advanced Materials Science (RCAMS), King Khalid University, P.O. Box 9004, Abha 61413, Saudi Arabia; 4 Department of Physics, Faculty of Science, King Khalid University, P.O. Box 9004, Abha 61413, Saudi Arabia; 5 Department of Mechanical Engineering, Mehran University of Engineering and Technology, Jamshoro 76090, Pakistan; 6 Department of Chemistry, College of Science, King Khalid University, P.O. Box 9004, Abha 61413, Saudi Arabia; 7 Department of Physics, Faculty of Science, King Abdulaziz University, Jeddah 21589, Saudi Arabia; 8 Physics and Engineering Mathematics Department, Faculty of Electronic Engineering, Menoufia University, Menouf 32952, Egypt; 9 Research Center for Nano-Materials and Energy Technology (RCNMET), School of Engineering and Technology, Sunway University, Bandar Sunway, Petaling Jaya 47500, Malaysia; 10 Department of Economics, Management, Industrial Engineering and Tourism (DEGEIT), University of Aveiro, 3810-193 Aveiro, Portugal

**Keywords:** phase hange materials (PCM), organic PCM (RT44HC), polyethylene glycol (PEG), activated carbon macroparticles (ACMPs)

## Abstract

Low thermal conductivity is the major obstacle for the wide range utilization of phase change materials (PCMs), especially organic PCMs, for most practical applications in thermal engineering. This study investigates the potential of enhancing the charging and discharging rates of organic PCM (RT44HC) by introducing polyethylene glycol (PEG) and activated carbon macroparticles (ACMPs). Different concentrations of PEG and ACMPs ranging from 0.3 wt% to 2 wt% were tested separately. The optimized concentrations found were used as dual reinforcements to attain the highest possible thermal conductivity. The specimens were tested for a complete charging–discharging cycle using an improvised thermal apparatus. Use of ACMP alone resulted in a minimal reduction in complete charging–discharging time due to the settlement of ACMPs at the bottom after 2–3 heating–cooling cycles. However, the addition of PEG with ACMPs exhibited a reduction in charging–discharging time due to the formation of a stable dispersion. PEG served as a stabilizing agent for ACMPs. The lowest charging–discharging time of 180 min was exhibited by specimens containing 1 wt% PEG and 0.5 wt% ACMPs which is 25% lower compared to bare PCM.

## 1. Introduction

Natural resources are getting depleted day by day as the never-ending appetite for energy is being satisfied. Mainly due to the lower cost of using non-renewable sources in the energy sector, mass exploitation and application of them have resulted in an unbalance in the ecosystem, climate change, various cases of pollution, and ozone layer deterioration [1,2,3]. That is why in recent times more renewable energy plants that are essential for sustainable development are installed. In this context, thermal energy storage systems hold great potential in reducing the dependence on fossil fuels, utilizing abundant solar energy, and other green energy sources [4,5,6].

One of the most promising and innovative ways for latent heat storage is using phase change materials (PCMs) that can store and release energy without harmful consequences to the environment. PCMs are regarded as environmentally friendly [7]. During the phase change process, PCMs can store and release a large amount of energy. Other unique characteristics include low cost, great heat capacity, high density, thermal and chemical stability, non-corrosiveness, non-segregating, non-toxic, and low or no supercooling [8,9]. However, low thermal conductivity and leakage are the major drawbacks of PCMs for practical applications. To overcome the inferior thermal conductivity of organic PCMs, dispersing thermally conductive particles is one of the common and reliable solutions [10].

Highly conductive metallic or non-metallic particles are introduced into PCMs to function as linkers for heat transfer enhancement. There are several research studies on the effect of particles on the thermal properties of PCMs based on size, shape, type of materials, and most importantly, concentration [11]. The thermal conductivity of pure PCMs is improved by 20% to 100% by the introduction of these additives. Wong et al. [12] demonstrated that the addition of nanoparticles (NPs) to salt hydrate-based PCM enhanced their thermal conductive properties by retarding phase segregation and subcooling. Graphene nanoplatelets (GNPs) and carbon nanotubes (CNTs) were applied to PCMs with WinSperse3050 as a dispersion stabilizer. With the dispersion of 5% GNPs in paraffin PCM, the thermal conductivity of the PCM was enhanced by 37.1% compared to the pristine PCM. Moreover, the melting rate, the freezing rate, and the thermal stability were also improved by the insertion of CNTs and GNPs [13].

Shahsavar et al. [14] carried out numerical and experimental studies on the impact of concentration and temperature of NPs on the thermal performance and viscosity of paraffin-Fe_3_O_4_ mixture along with the usage of oleic acid as a dispersant. The results depicted that both viscosity and thermal conductivity were boosted with respect to the increased concentration of NPs while on the other hand, viscosity declined, and thermal conductivity rose as temperature increased. Benbrika et al. [15] used graphene nanoplatelets in combination with PCM using horizontal cylinders in a numerical analysis that described the solidifying and melting characteristics of GN. Findings demonstrated a reduction of 50% in solidification time and a small decline in melting time. However, on the other hand, there was also a dip in energy storage capacity.

Yang et al. [16] introduced boron nitride (BN) and graphene oxide (GO) into PEG to function as thermally conductive particles in an attempt to boost thermal conductivity and stability. It was found that the resultant composite PCM with 4% GO and 30% BN exhibited a thermal conductivity value of 3 W/m·K of thermal conductivity which is 10-fold greater than that of pristine PEG. Sun et al. [17] reported improvement in the thermal energy storage performance of paraffin PCM using nano graphite and nano coconut shell charcoal at nanocarbon concentrations of 0.02%, 0.06%, and 0.10% weight, respectively. PCM with 0.06% graphite NPs and with 2 wt% of oleic acid as dispersant revealed a 21% shortening of melting time compared to pure PCM.

Kalidasan et al. [18] synthesized polyaniline-cobalt NPs nanocomposite and dispersed the composite in paraffin PCM in different amounts (0.1, 0.5, 1, and 5%). The resultant PCM composite showed a 15.9% increase in latent heat of fusion and a 20.4% increase in thermal conductivity. Therefore, it can be concluded that over the last few decades, the two-step fabrication method was widely applied to PCMs for synthesizing PCMs with several types of supporting conductive materials by taking into consideration the issues of shape stability and thermal performance.

Although several studies were focused on carbon-based composite PCMs, there are relatively few works that utilize activated carbon macroparticles (ACMPs). ACMPs are thermally conductive, cheap, and readily available in the market. The use of PEG as a PCM is well known in the literature. However, the incorporation of PEG into PCM as a dispersion stabilizer is yet to be examined. Therefore, in this work, paraffin was used as a base material to which PEG and ACMPs were added to enhance the thermal conductivity for thermal energy storage applications.

## 2. Materials and Methods

There are two major methods being used for the synthesis of nanoparticles based PCMs, namely, one-step and two-step methods. If the synthesis and dispersion of nanoparticles in base PCM take place in a single step it is called one-step method. While in two-step method, the nanomaterial is first synthesized or purchased from market and then dispersed into base PCM. Most of the researchers used two-step method for the preparation of nanoparticle-based PCM [19]. Selection of PCM can depend on the ambient temperature where the thermal energy storage application is desired. As average daytime temperature of the region under investigation, Malaysia is around 28 °C, so PCMs with melting points around 42 to 50 °C has been considered for the present application [20]. However, the focus of this research is to investigate the effect of additives on thermal conductivity of PCM; therefore, the selection of PCM is independent of regional weather conditions. RT44HC has been selected for the current study.

### 2.1. Materials

All of the chemicals used in this research study were analytical grade, and they were used as received, with no purification. The activated carbon macroparticles (ACMPs) with an average diameter of 0.1 to 0.3 mm and a density of 500 kg/m^3^ were obtained from EvaChem Chemical Distributor Malaysia to be utilized as thermally conductive materials. Polyethylene glycol (PEG-435457), with a density of 1.006 g/mL was acquired from Aldrich Chemistry USA to be used as a surfactant. The solid paraffin wax for the base phase change material (PCM-RT44HC) with a density of 0.8 kg/L and a melting temperature of 41–44 °C was purchased from Rubitherm Phase Change Materials. Important physical properties of PCM-RT44HC are listed in Table 1 [21].

**Figure 1:**
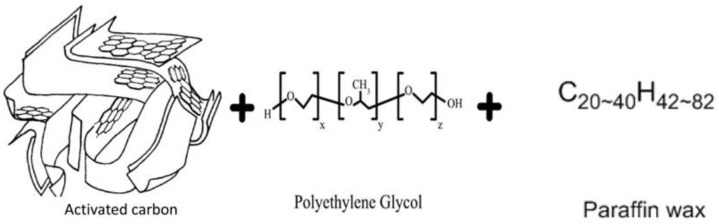
Molecular structure of ACMPs, PEG, and organic PCM [29].

Furthermore, from X-ray diffraction studies it is known that for thermally activated carbon, the structure is similar to that of ideal graphite. Within graphite, the Van der Waals forces keep apart the multiple layers of merged hexagons by approximately 0.34 nm [22,23,24]. PEG is made up of an O(CH_2_)_2_ monomer unit that is categorized by not only a polar oxygen atom but also a non-polar (CH_2_)_2_ group, and it gets easily dissolved in a wide range of polar or non-polar solvents by anionic chain transfer polymerization preparation technique [25,26]. Paraffin waxes comprise a mixture of straight n-alkane chains, C_n_H_2n+2_. The variation of n is within 12 to 28 while the melting temperature ranges between −10 and 61 °C, making them the most common type of organic PCMs [27,28]. Molecular structures of ACMPs, PEG, and organic PCM are shown in Figure 1.

### 2.2. Instruments

The thermal performance of the macrofluid was evaluated using the facilities in the Cell Solar Testing Laboratory located within Wisma R&D, UMPEDAC, Malaysia. An HR-250AZ ultrasonic weighing balance was used to prepare multiple mixtures with different proportions of the base PCM and the additives. Cylindrical plastic sample cups of (H 7 cm × dia Ø 4 cm) size were used as containers for macro-enhanced PCMs. A hot plate (company: prosperity biotech, Shandong, China) was applied for heating and magnetic stirring of the mixtures. A T1108 temperature meter was applied for recording the PCM heating and cooling After synthesizing, the nanoparticle-based PCM was characterized for its microstructural features, phase transition properties, and thermal and chemical stabilities. Microstructural analysis was carried out using a high-resolution scanning electron microscope (SEM). Phase transition phenomena within the materials were studied using a differential scanning calorimeter (DSC) within the temperature range from 25 °C to 80 °C at a ramp rate 1 °C/min. Chemical stability of the materials was confirmed through Fourier transform infrared (FTIR) spectra recorded in the wave number range of 4500 cm^−1^ to 400 cm^−1^ under static atmosphere.

### 2.3. Preparation

The total weight of each sample was set exactly at 15 g wherein both the particle (ACMPs) and the surfactant (PEG) amounts were based on the PCM quantity. For instance, if 14.706 g of PCM were to be used as the base material, for 1% weight of PEG and 1% of ACMPs, 0.147 g, and 0.147 g will be, respectively, added to the PCM so that the total weight would be 15 g. The experiment was divided into 03 sections: ACMPs addition, PEG addition, and the addition of both PEG and ACMPs to the PCM for achieving clear objectives and results. There were multiple parameters for comparison where each cup was run 3 times for stabilized results. ACMPs were inserted at different weight fractions (0.3%, 0.5%, and 1%) and PEG was mixed at 0.5%, 1%, and 2% of the total materials, namely, PCM + ACMPs, PCM + PEG and PCM + ACMPs + PEG. It is worth mentioning here that the specimens with various concentrations have been prepared and investigated to find out the optimum concentration of additives in PCM. The optimum concentration was based on the minimum to no precipitation and percent increase in thermal conductivity which was co-related with other parameters, i.e., enthalpy and possible chemical reactions after thermal cycles. 

**Figure 2:**
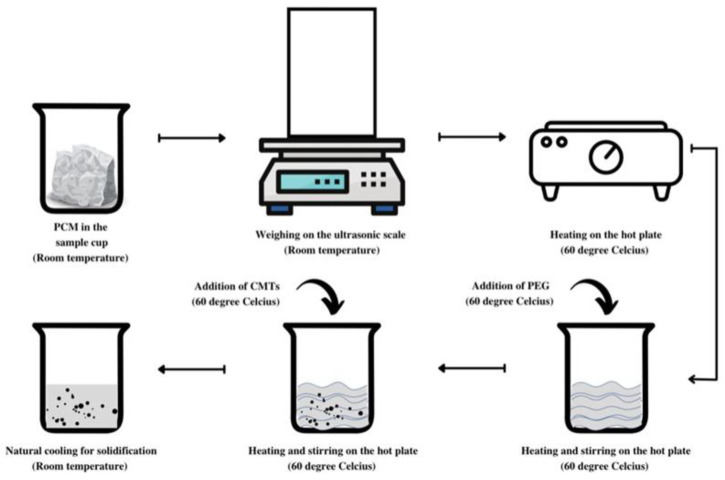
A schematic diagram for the preparation of macro-enhanced PCM.

Figure 2 shows the schematic illustration of the sample preparation method. Firstly, the solid paraffin was weighed and placed in the sample cup. The hot plate was put on and the temperature was allowed to reach 60 °C and the sample cup was heated until all the PCM was melted. Subsequently, 0.5%, 1%, or 2% of PEG were mixed with paraffin under stirring at 500 rpm and with continuous heating at 65 °C for about 10 min. Then, 0.3%, 0.5%, or 1% of ACMPs were added into the mixture and dispersed again by magnetic stirring at 900 rpm and with continuous heating at 65 °C for about 15 min. Following this step, the sample cup was placed at room temperature for solidification.

### 2.4. Data Logging the Charging and Discharging Rates

**Figure 3:**
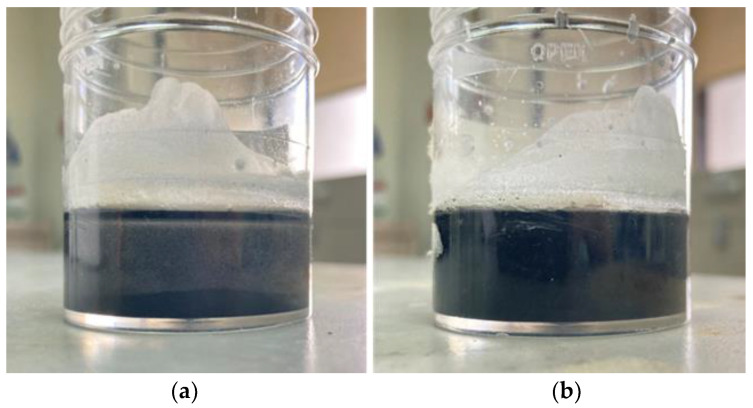
Images showing the dispersion stability of PCM material with (**a**) ACMPs only and with (**b**) ACMPs + PEG after 2nd cycle.

When the mixture (PCM+ additives) became a complete solid, the sample cup was connected with thermocouples at the top surface. After that, the sample cup is placed at 60 °C using a hot plate (constant temperature) with the running temperature meter. Cycle temperature was recorded every 30 s within the temperature range of 26 °C and 48 °C. By the time the whole mixture was melted, and the surface temperature reached 46 °C, the sample cup was immediately taken from the hot plate to a neutral environment for the natural discharging process. Cooling period was recorded until the surface temperature was equivalent to the room temperature wherein the mixture was totally solidified, and then, the cycle was repeated once again. Though the temperature was recorded from 5th cycle in each run, only the results from the 6th cycle onwards were discussed in this study as visual stability was fully obtained only after the 2nd cycle. This means that no further precipitation of ACMPs at the bottom was noticed after the 2nd cycle as can be observed in Figure 3.

## 3. Results and Discussion

**Figure 4:**
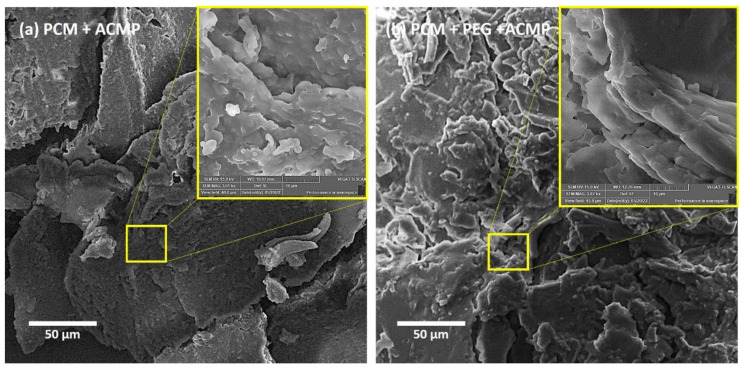
SEM images of phase change material (PCM) samples containing optimum concentration of (**a**) paraffin wax and activated carbon macroparticles (ACMPs) and (**b**) paraffin wax + polyethylene glycol (PEG) + activated carbon macroparticles (ACMPs).

The nanoparticle-based PCM was characterized using SEM (the term microscopy is involved in SEM, and we need not add it once again), FTIR spectroscopy, and DSC analysis. SEM provides vital information on the morphology and homogeneity of the material. The scanning electron micrographs of RT44HC + AC and RT44HC + PEG + AMCPs are shown in Figure 4. The close-packed structure of nanoparticle-based PCM wherein AMCPs and PEG were homogeneously distributed on the rough and porous surface of RT44HC was seen in the SEM images. It is worth mentioning here that the samples were heated to make a thin tablet and fixed on the aluminum stud for SEM analysis. Furthermore, the gold coating has been performed to create an electrical path. The voltage chosen for SEM was 15 Kv stage to electron gun distance of 7 mm for all the samples.

### Heating and Cooling Performance

**Figure 5:**
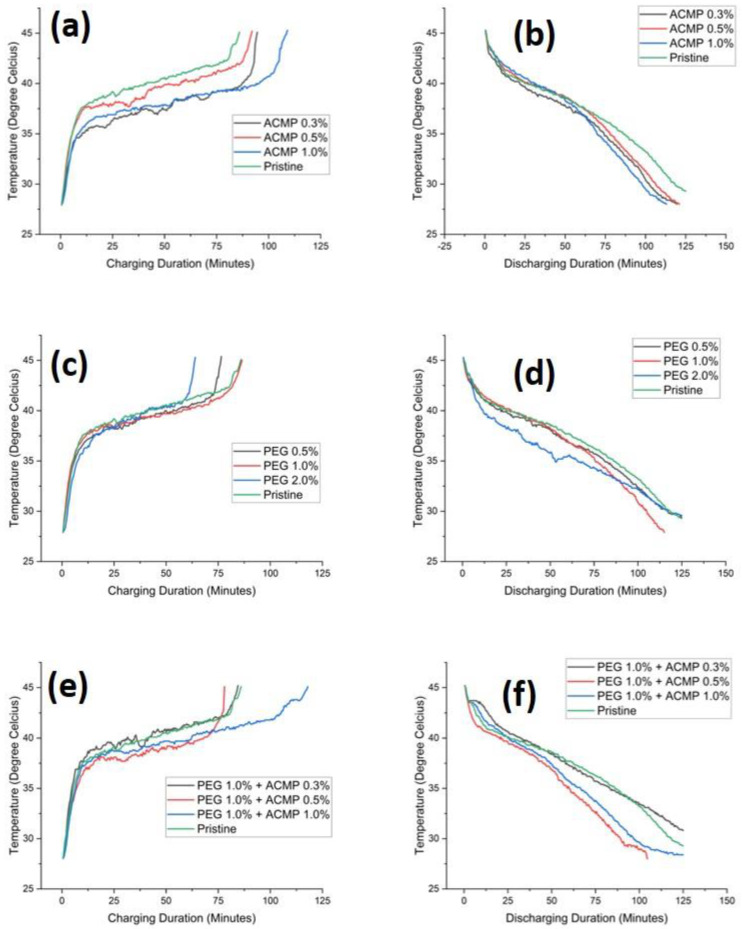
Charging and discharging rates of PCM mixed with different weight ratios of (**a**,**b**) ACMPs 0.3%, 0.5%, and 1%, (**c**,**d**) PEG 0.5%, 1%, and 2%, and (**e**,**f**) ACMPs 0.3%, 0.5%, and 1% at PEG 1%.

Charging and discharging curves of pure PCM, as well as PCM with additives, were recorded to make a direct comparison of the individual and combined effects of ACMPs and PEG. Experiments were conducted for each amount of the additive and the best amount of PEG was selected to optimize the concentration of ACMPs to be added to the paraffin wax (base PCM). The time vs. temperature behavior of specimens under observation are shown in Figure 5a–f. The details extracted from Figure 5 are presented in Table 2. It is worth mentioning that five heating–cooling cycles were recorded in each case and the curves for the sixth cycle onward were presented to avoid any errors related to the formation of air bubbles and precipitation. It is worth mentioning here that a total of 10 charging–discharging cycles have been conducted after the fifth cycle and the observed curves were found to be identical. This is because of no change in the chemical composition of PCM. The characteristic phase change properties remain the same.

With the incorporation of ACMPs in PCM, the overall charging–discharging time decreased. However, the change is not so prominent. When 0.3 wt% ACMPs (Figure 5a) were added to PCM, a rapid gain in temperature has been observed until 34 °C which may be called the pre-sensible heat. The rate of temperature decrease after that indicated the start of the change in phase and may be referred to as the post-sensible heat. Complete liquefaction was observed after around 90 min that marked the onset of a rapid gain in temperature rate. With specimens containing 0.5 wt% ACMPs, the same trend was observed. An onset of phase change and post-sensible heating was observed until 40 °C and complete melting was observed after around 87 min. Further increase in the concentration of ACMPs to 1 wt% in PCM reduced the onset of phase change temperature to around 36 °C. Complete liquefaction of PCM was observed after 105 min which marked the onset of phase change and an increase in the rate of temperature. On the other hand, the onset temperature of phase change for pure PCM was between 36–37 °C. Complete liquefaction was observed after around 80 min which is the shortest time. This implies that the incorporation of ACMPs alone did not increase the thermal conductivity, but rather reduce it. This is due to the inability of ACMPs to for stable suspension of PCM. It was observed that most of the ACMPs precipitated out of the PCM after the third heat–cooling cycle. The discharging behavior of the PCM incorporated with ACMPs is shown in Figure 5b. The discharging time of pure PCM was the maximum compared to PCMs incorporated with ACMPs. This behavior can be correlated with the effect of reduction in pre-sensible heat, which reduced the energy storage with the incorporation of ACMPs.

The charging and discharging behavior of PCM incorporated with PEG are, respectively, shown in Figure 5c,d. The incorporation of PEG in PCM did reduce the overall charging and discharging time. A minimum charging–discharging time of 201 min was observed for specimens containing 1 wt% PEG in PCM. Further increase in PEG did not show any increase in overall charging–discharging time. So, the concentration of 1 wt% PEG in PCM was selected for dual reinforcement. In terms of discharging time, pure PCM took the longest time whereas, the specimens with 1 wt% of PEG in PCM showed the lowest discharging time.

The charging–discharging behavior of PCM incorporated with both ACMPs and PEG where the concentration of PEG was kept at 1 wt% relative to PCM shown in Figure 5e,f. The onset temperature of phase change was observed to be the same for all the amounts of ACMPs, namely, 0.3 wt%, 0.5 wt%, and 1 wt% in PCM-PEG mixtures. With the incorporation of 0.3 wt% ACMPs in the PCM-PEG solution, the time for complete liquefaction reduced a bit. The minimum time for complete liquefaction was shown by the specimens containing 0.5 wt% ACMPs in PCM-PEG solution. Further increase in ACMPs increased the liquefaction temperature due to precipitation. On the other hand, the minimum discharging time shown by the specimens containing 0.5 wt% ACMPs in PCM-PEG solution which infers that the best concentration of ACMPs and PEG in PCM (paraffin wax) were 0.5 wt% and 1 wt%, respectively. As PEG is a good dispersing agent, surface modification of ACMPs with PEG increased the thermal conductivity of the PCM (associated with a reduction in the overall charging–discharging cycle) by the formation of proper dispersion and the stable slurry of ACMPs in PCM.

**Figure 6:**
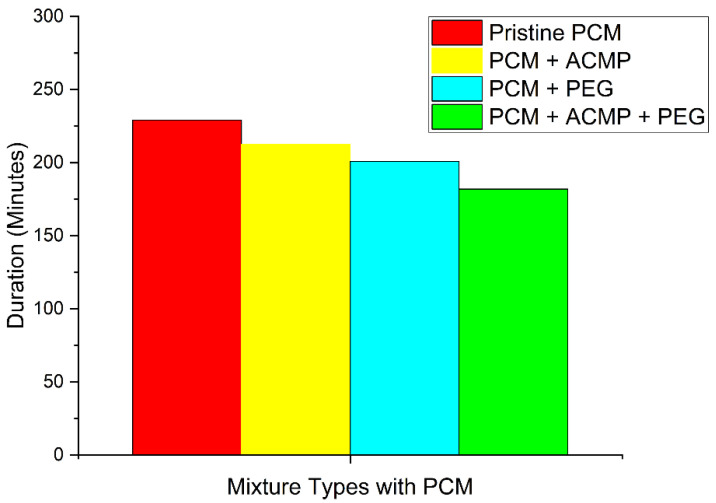
Comparison of total time taken for charging and discharging by each method with that of pristine PCM.

Comparative overall charging–discharging time of pure PCM, the optimized concentration of ACMPs in PCM (0.3 wt%), the optimized concentration of PEG in PCM (1 wt%), and optimized concentrations of both the ACMPs and PEG, i.e., 0.5 wt% and 1 wt% in PCM, respectively, are shown in Figure 6. The overall trend is the decrease in time with the incorporation of optimum concentrations of individual and combined reinforcements, namely, ACMPs and PEG. Minimum overall charging–discharging time was observed for specimens containing both the ACMPs and PEG. This is due to the formation of the stable slurry of ACMPSs in the PCM caused by PEG.

**Figure 7:**
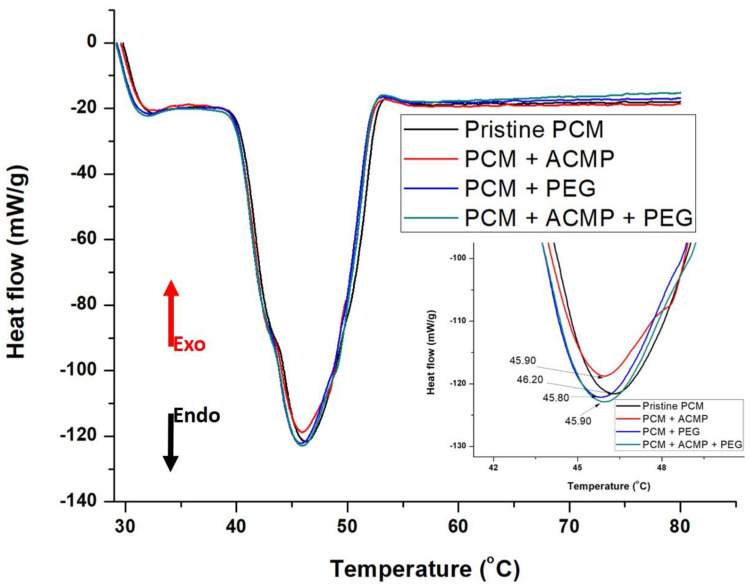
DSC curves of pristine PCM and PCM with additives with optimum compositions.

DSC curves of specimens containing optimized amounts of individual and combined additives were recorded to investigate the thermal properties of the PCM. DSC pattern of the specimens containing optimized concentrations are shown in Figure 7. The detailed parameters deduced from the plots were summarized in Table 3. Very insignificant to no change in onset temperature was observed which is in line with the observation presented in Figure 5a,c,e. Furthermore, no change in recrystallization temperature was observed with the incorporation of ACMPs and PEG in PCM. However, a reduction in enthalpy was observed with the incorporation of ACMPs in PCM. As enthalpy is directly related to the energy storage capacity of the PCM, a reduction in enthalpy leads to a reduction in the energy storage ability of the PCM. 

In comparison, the addition of ACMP reduces the latent heat by 7.87 J/g relative to pristine PCM. Hence, it can be deduced that individual addition of ACMP has a greater change on the latent heat. It can be explained using the second law of thermodynamic equations: (∆*H* = ∆*U* + *P*∆*V*, ∆*U* = *T*∆*S* – *P*∆*V*) [30]. An increase in entropy (∆*S* > 0) occurs with the addition of particles which increases chaos inside the mixture. This disturbance in the system leads to an increase in the internal energy of the system. As ∆*H* = ∆*U* + *V*∆*P*, hence enthalpy of the system increases. With the addition of particles, the second term (*P*∆*V*) in the internal energy becomes greater than the first term (*T*∆*S*), thereby reducing the internal energy of the system. The enthalpy of the system decreases due to a decrease in internal energy.

On the other hand, PEG did not reduce the latent heat of PCM. However, the combined inclusion of ACMP and PEG reduces the latent heat by just 1.53 J/g which is a 0.1% reduction. This effect can be correlated with proper dispersion and the formation of a stable suspension of particles. It is worth mentioning here that for the DSC test, 1 g of weight has been used for all the specimens.

The melting temperature of pristine PCM, PCM + ACMP, PCM + PEG, and PCM + ACMP + PEG is 45.90 °C, 45.90 °C, 45.80 °C, and 46.20 °C, respectively, where onset and offset temperatures are listed in Table 3. It can be concluded that the addition of ACMP and PEG has no significant effect on the melting point of PCM.

**Figure 8:**
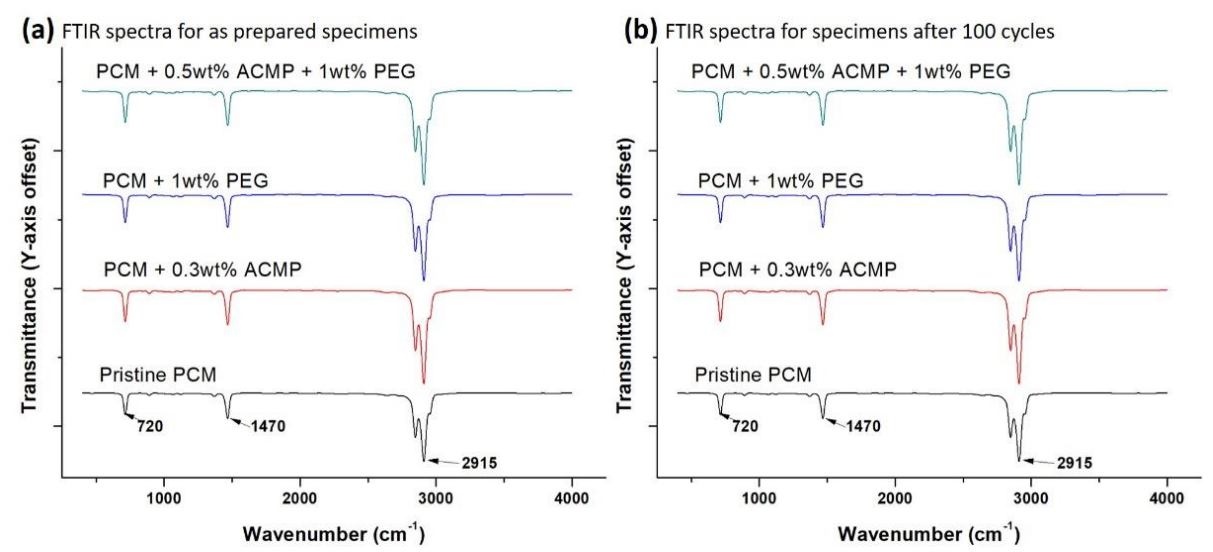
FTIR spectra of samples with optimum compositions (**a**) before and (**b**) after 100 consecutive thermal cycles.

FTIR study was conducted on PCMs to evaluate the stability of the formulation as well as to identify any of the chemical reactions that may have taken place. Figure 8a,b shows the FTIR pattern of as-prepared specimens and specimens after 100 after 100 charging–discharging cycles, respectively, in the range of 400–4000 cm^−1^. Figure 8a shows the FTIR spectrum of pristine RT44HC (as received) and doped with AC and PEG (individual and combined doping), whereas Figure 8b shows the FTIR spectra of specimens after 100 charging–discharging cycles.

The characteristic peaks of paraffin wax have been picked up at 720 cm^−1^, 1470 cm^−1^, and 2915 cm^−1^ which corresponds to CH_2_ group rocking, the deformation of CH_3_ and CH_2_ groups, and CH_2_ group symmetric stretching vibration, respectively. No peaks for ACPMs and PEG have been observed. This may be due to the low concentration of additives in the PCM. Furthermore, nearly identical FTIR spectra can be observed in Figure 8b which has been performed after 100 cycles. It is evident that no new peak formation occurs in the prepared composite after 100 cycles and hence, the chemical composition has not been altered.

## 4. Conclusions

In this study, the individual and combined effects of ACMPs and PEG have been investigated for increased thermal conductivity of pristine PCM. Different concentrations of PEG and ACMPs ranging from 0.3 wt% to 2 wt% were tested separately. The optimized concentrations found were used as dual reinforcement to attain the highest possible thermal conductivity. The SEM was employed to examine the surface morphology and homogeneity of the PCMs with additives, namely, ACMPs and PEG, and an improvised setup has been fabricated to study the charging–discharging behavior of the PCMs to optimize the concentration of additives. Finally, DSC and FTIR studies have been performed to calculate the change in enthalpy and the stability of the specimens after a hundred charging–discharging cycles, respectively. An overall increase of 25% in thermal conductivity was observed when optimum concentrations of both the ACMPs and PEG have been studied, which is due to the synergistic effect of both additives. Further increase in concentration did not increase the thermal conductivity due to the excessive settling of particles at the bottom.

## Figures and Tables

**Table 1:** Properties of RT44HC phase change material (PCM).

Property	Value
Melting temperature (°C)	41–44
Heat storage capacity, ±7.5% (kJ/kg)	250
Specific heat capacity (kJ/kg·K)	2
Density of solid PCM at 25 °C (kg/L)	0.8
Density of liquid PCM at 25 °C (kg/L)	0.7
Heat conductivity (W/m·K)	0.2
Volume expansion (%)	12.5
Flash point (°C)	>180
Maximum operation temperature (°C)	70

**Table 2:** Enhancement of charging and discharging rates of phase change material (PCM) with additives.

Parameter	Pristine PCM	PCM + ACMPs	PCM + PEG	PCM + ACMPs + PEG
Composition		0.3 wt% ACMPs	1 wt% PEG	0.5 wt% ACMPs, 1 wt% PCM
Charging rate (°C/min)	0.20	0.18	0.20	0.22
Discharging rate (°C/min)	0.12	0.14	0.15	0.16
Enhancement for charging	-	−7%	−1%	10%
Enhancement for discharging	-	19%	25%	37%

**Table 3:** Detailed thermal parameters of samples with optimum compositions.

S. No	Sample Details	Onset Temperature of Phase Change (°C)	Offset Temperature of Phase Change (°C)	Point of Reaction	Enthalpy (J/g)
1	Bare PCM	40.10	54.20	−59.05 mW at 41.7 °C	−259.76
2	PCM + ACMPs	40.10	53.80	−60.51 mW at 41.6 °C	−251.89
3	PCM + PEG	39.70	53.70	−56.67 mW at 41.3 °C	−259.52
4	PCM + ACMPs + PEG	39.80	54.00	−58.73 mW at 41.4 °C	−258.23

## Data Availability

The scientific data will be furnished on request.
